# Supplementary material for: The HisCl1 histamine receptor acts in photoreceptors to synchronize Drosophila behavioral rhythms with light-dark cycles
Source: Nat Commun. 2019 Jan 16;10:252. doi: 10.1038/s41467-018-08116-7 (PMC6335465; doi:10.1038/s41467-018-08116-7)
Supplement: Supplementary file 1 — Supplementary Information [file 41467_2018_8116_MOESM1_ESM.pdf]

Supplementary Information

The HisCl1 histamine receptor acts in photoreceptors to synchronize *Drosophila* behavioral rhythms with light-dark cycles

Alejevski et al.

Sup. Fig. 1, top

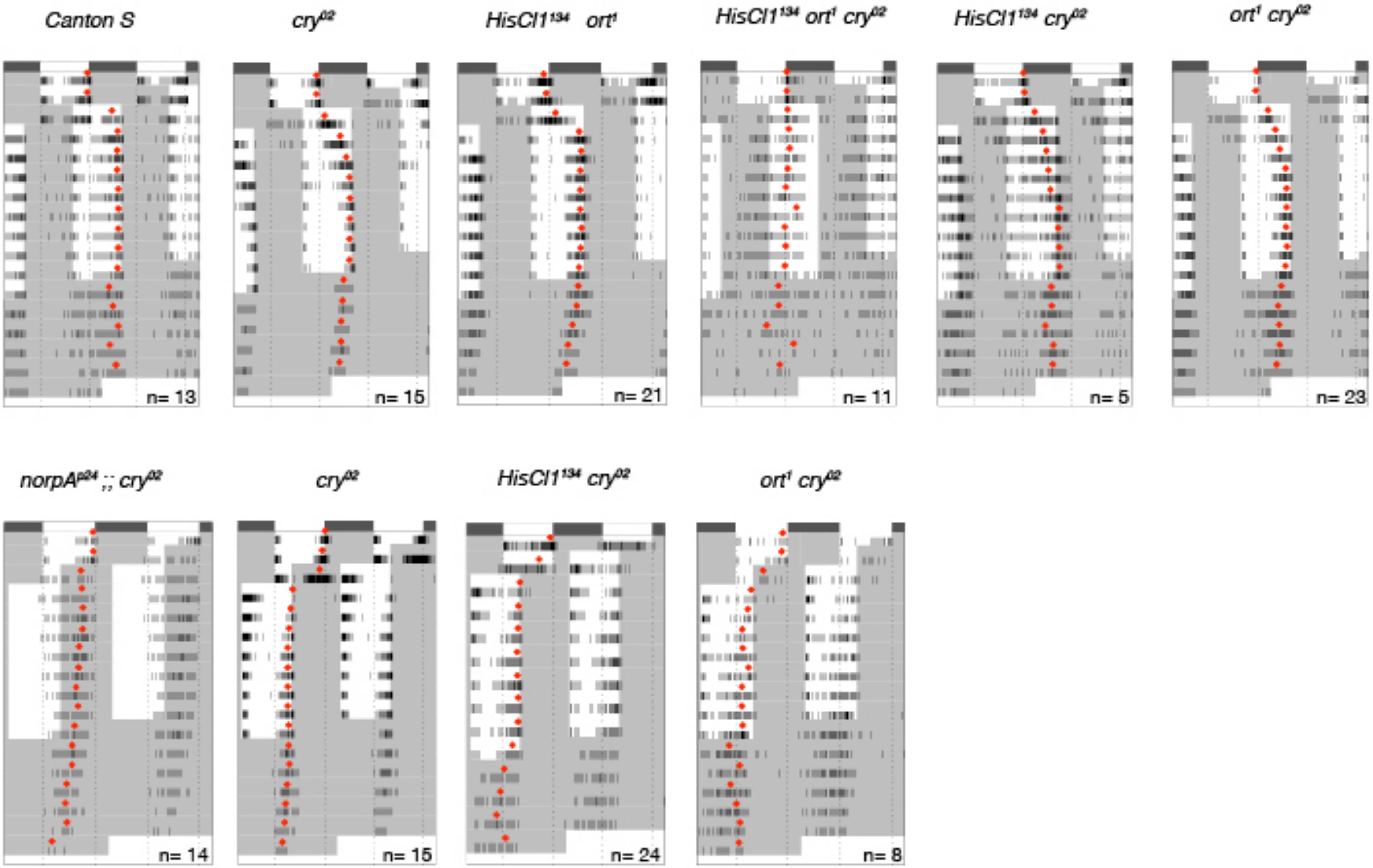

Low light

Sup. Fig. 1, bottom

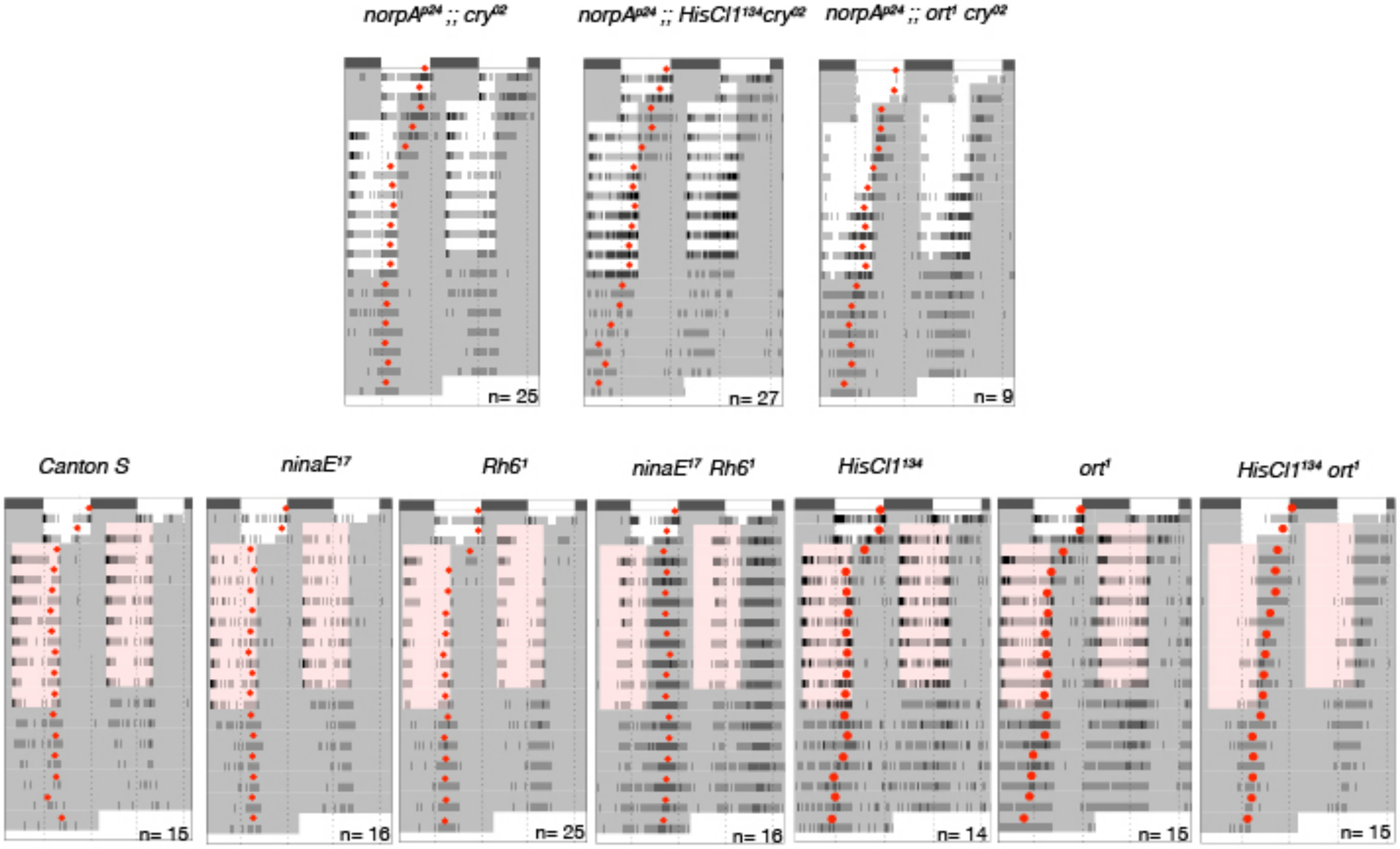

Supplementary Fig. 1: Role of HisCl1 and Ort in the Cry-independent synchronization of the circadian clock. Top first row. Average double plotted actograms of flies exposed to an 8h delay of the LD cycle. Flies were initially entrained with both LD and Temperature Cycles (TC: 25-20°C). T°C was kept constant (25°C) from the beginning of the day 2 light phase until the end of the experiment. Between days 2 and 3, the dark phase was lengthened by 8h. Thus, flies were exposed to a novel delayed LD regime for 8 cycles followed by constant darkness (6 DD cycles). Wild type flies as well as flies with no Cry or no histamine receptors synchronize with phase delayed LD cycles. Flies with no Cry and no histamine receptors do not synchronize, whereas flies with no Cry and only Ort or only HisCl1 do synchronize. **Top second row.** Average double plotted actograms of flies exposed to an 8h advance of the LD cycle. The experimental design is as described in the Figure 1 legend, except that low light (see Methods) is used for the shifted LD cycle. In the absence of Cry, each one of the two histamine receptors allows NorpA-dependent synchronization. **Bottom first row**. Average double plotted actograms of flies exposed to an 8h advanced LD cycle. The experimental design and actograms are as described in legends of Figure 1. Each of the two histamine receptors supports synchronization in the absence of Cry and NorpA. **Bottom second row.** Average double plotted actograms of flies exposed to an 8h advanced RD cycle. The experimental design and actograms are as described in legends of Figure 4. Each of the Rh1 and Rh6 rhodopsin supports synchronization with RD cycles whereas the absence of both Rh1 and Rh6 prevents it (see [1]). Each of the two histamine receptors supports synchronization with RD cycles, whereas the absence of both Ort and HisCl1 prevents it.

Sup. Fig. 2

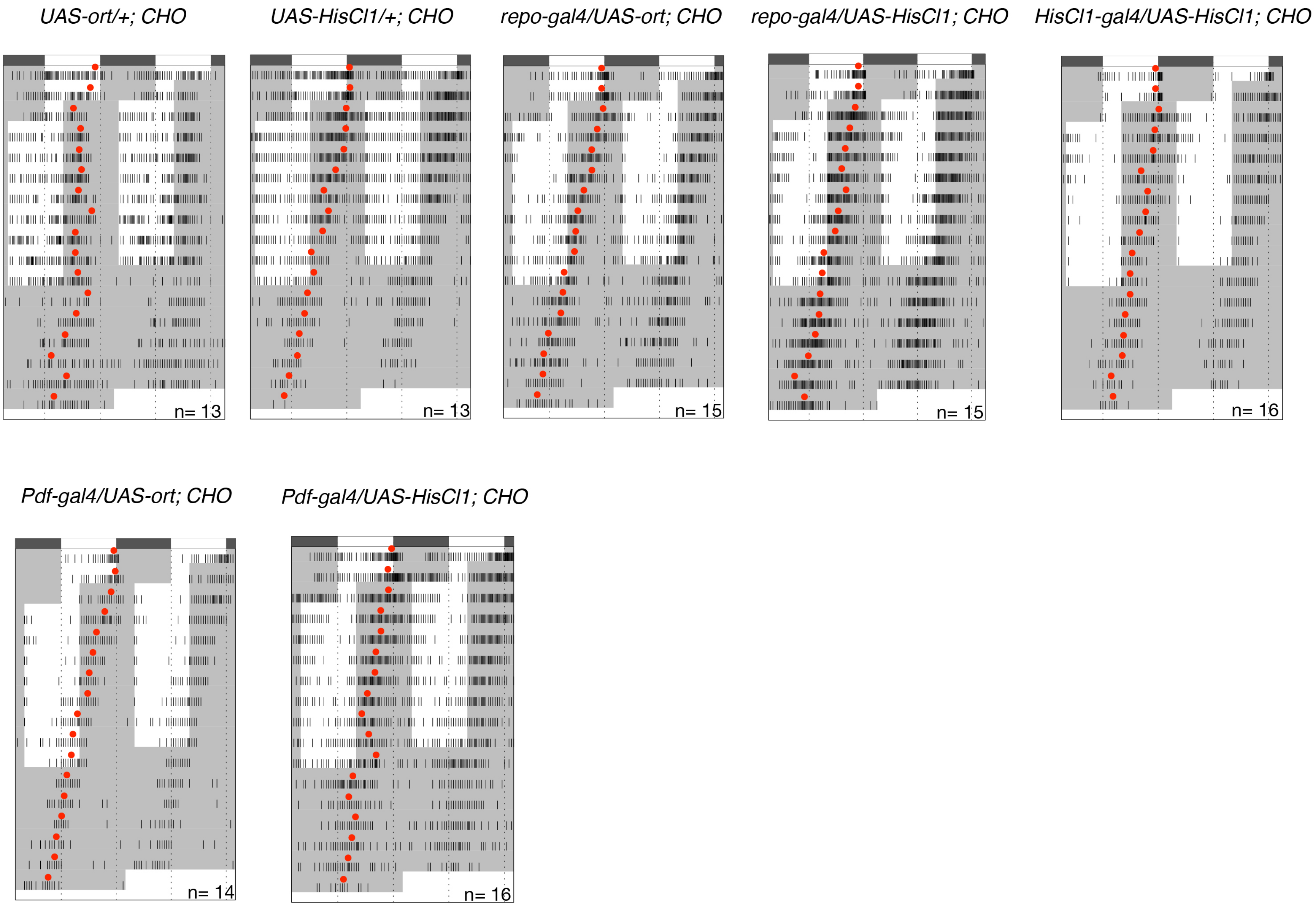
UAS-ort/+; CHO
n= 13
UAS-HisCl1/+; CHO
n= 13
repo-gal4/UAS-ort; CHO
n= 15
repo-gal4/UAS-HisCl1; CHO
n= 15
HisCl1-gal4/UAS-HisCl1; CHO
n= 16
Pdf-gal4/UAS-ort; CHO
n= 14
Pdf-gal4/UAS-HisCl1; CHO
n= 16

Supplementary Fig. 2: Expression of Ort and HisCl1 in glial cells or in PDF neurons do not support synchronization of the clock. Average double plotted actograms of flies exposed to an 8h advance of the LD cycle. The experimental design is as described in Figure 1 legend. **Top**. Expression of either *ort* or *HisCl1* in most glial cells of the brain *(repo-gal4)* of *CHO* mutants does not support synchronization. *HisCl1* expression in the lamina epithelial glial cells showing expression of *HisCl1-gal4* [2] does not rescue synchronization. Although two copies of a different *HisCl1-gal4* [3] did show very faint expression of GFP reporter in inner photoreceptors (Fig. 3), no behavioral rescue was observed when one copy was used to drive *HisCl1* expression (not shown), likely because of the very weak expression in photoreceptors. **Bottom**. No behavioral rescue with either *ort* or *HisCl1* expression in the PDF-expressing clock neurons.

Sup. Fig. 3

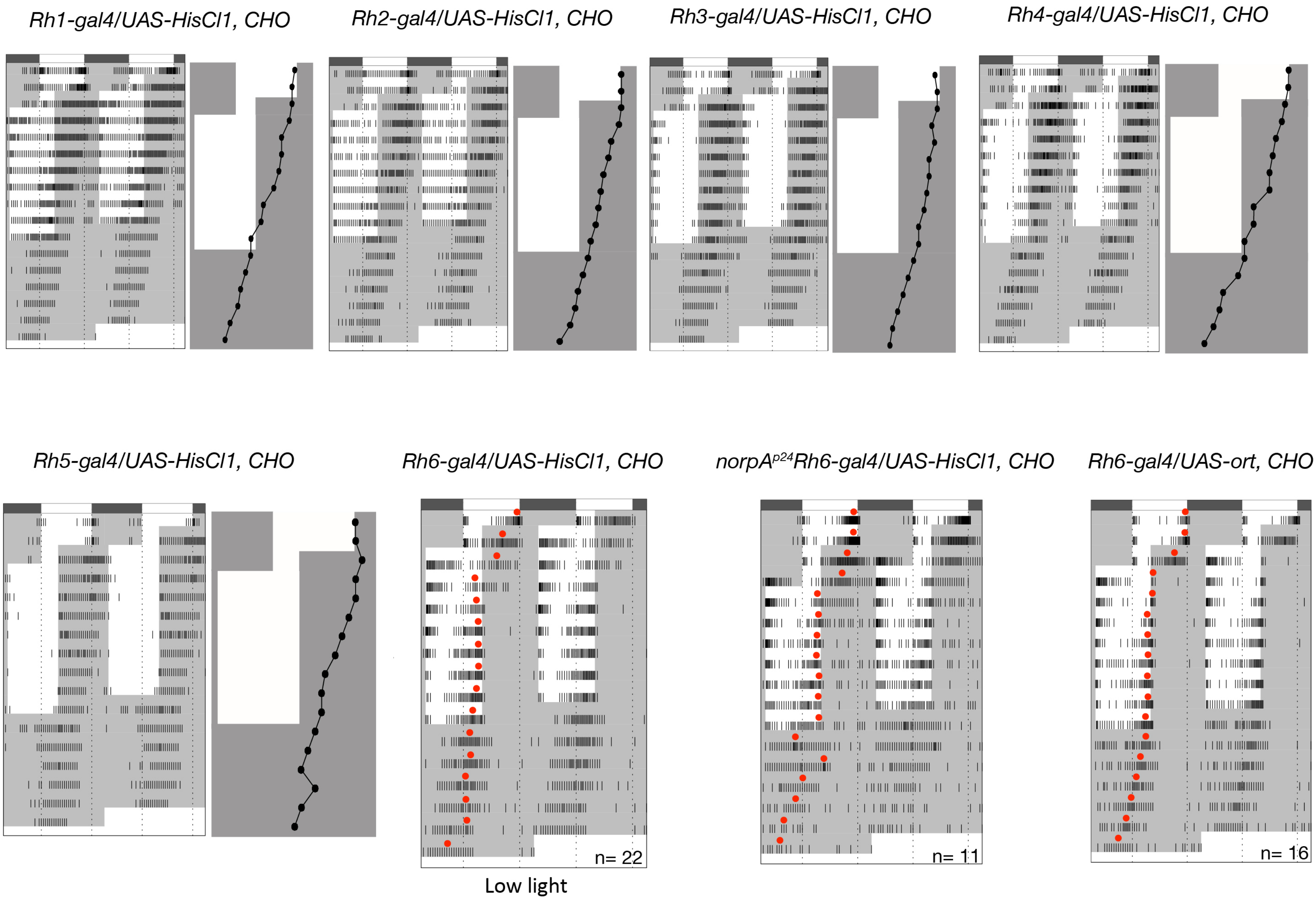
Rh1-gal4/UAS-HisCl1, CHO
Rh2-gal4/UAS-HisCl1, CHO
Rh3-gal4/UAS-HisCl1, CHO
Rh4-gal4/UAS-HisCl1, CHO
Rh5-gal4/UAS-HisCl1, CHO
Rh6-gal4/UAS-HisCl1, CHO
norpA^{p24}Rh6-gal4/UAS-HisCl1, CHO
Rh6-gal4/UAS-ort, CHO
n= 22
n= 11
n= 16
Low light

Supplementary Fig. 3: HisCl1 expression in Rh6 photoreceptors but not in other photoreceptors allows synchronization. Average double plotted actograms of flies exposed to an 8h advance of the LD cycle, with corresponding phase plots. The experimental design and actograms are as described in Figure 1 legend, except for the bottom left actogram where low light was used instead of standard high light for the advanced LD cycle (see Methods) **Top**. Expressing *HisCl1* with *gal4* drivers specific for Rh1-5 photoreceptors does not support entrainment. **Bottom**. HisCl1 expression in Rh6 photoreceptors supports entrainment through both the NorpA-dependent pathway (left) and the NorpA-independent pathway (center). Targeting *ort* expression in Rh6 cells rescues synchronization, indicating that *ort* can replace *HisCl1* function in photoreceptors (right).

Sup. Fig. 4

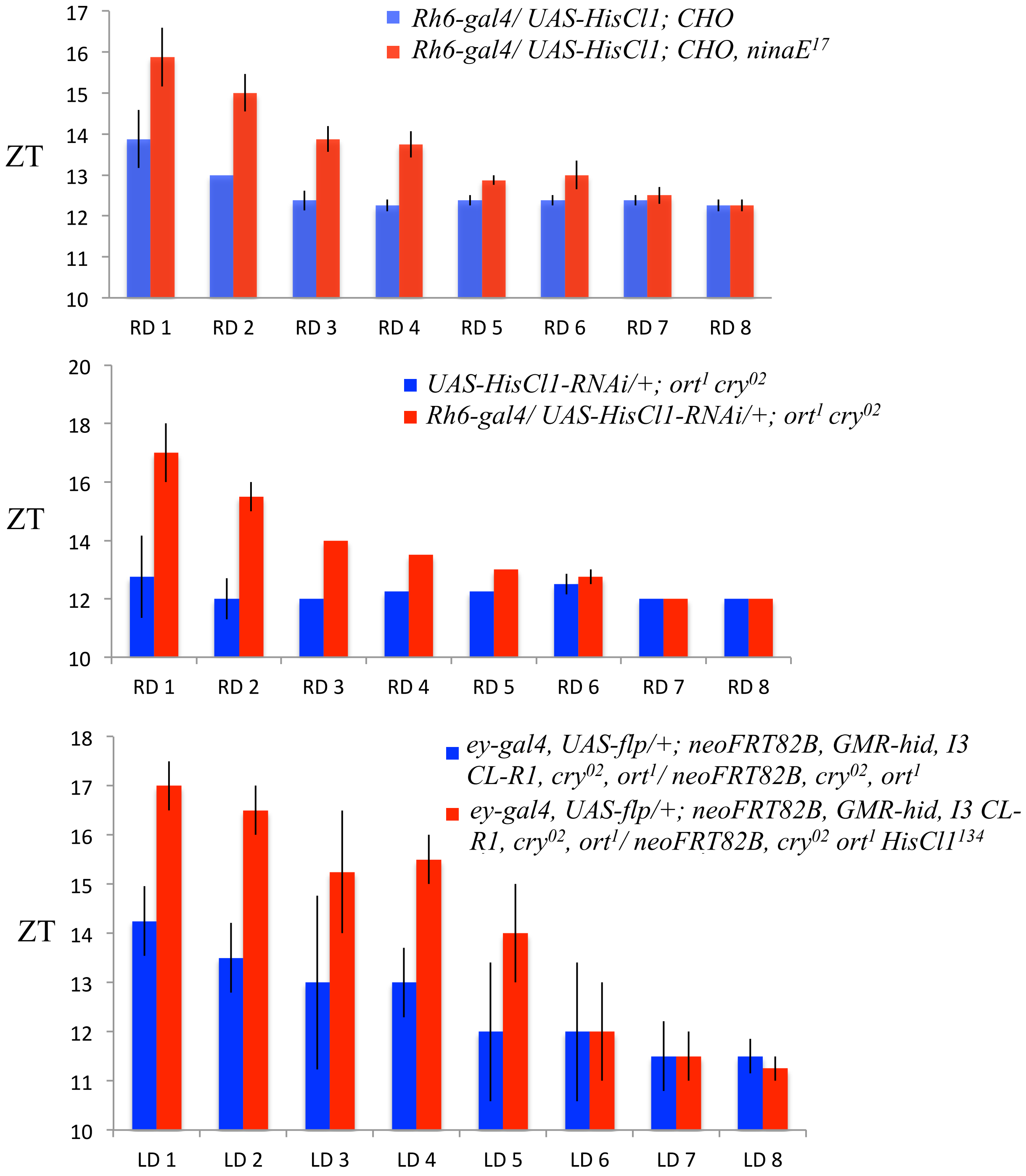
Rh6-gal4/ UAS-HisCl1; CHO
Rh6-gal4/ UAS-HisCl1; CHO, $ninaE^{17}$
ZT
17
16
15
14
13
12
11
10
RD 1
RD 2
RD 3
RD 4
RD 5
RD 6
RD 7
RD 8
UAS-HisCl1-RNAi/+; ort^{1} cry^{02}
Rh6-gal4/ UAS-HisCl1-RNAi/+; ort^{1} cry^{02}
ZT
20
18
16
14
12
10
RD 1
RD 2
RD 3
RD 4
RD 5
RD 6
RD 7
RD 8
ey-gal4, UAS-flp/+; neoFRT82B, GMR-hid, I3 CL-R1, cry^{02}, ort^{1}/ neoFRT82B, cry^{02}, ort^{1}
ey-gal4, UAS-flp/+; neoFRT82B, GMR-hid, I3 CL-R1, cry^{02}, ort^{1}/ neoFRT82B, cry^{02} ort^{1} $HisCl1^{134}$
ZT
18
17
16
15
14
13
12
11
10
LD 1
LD 2
LD 3
LD 4
LD 5
LD 6
LD 7
LD 8

Supplementary Fig. 4: Quantification of synchronization speed. Quantification of synchronization with an 8h advanced RD (top, center) or LD (bottom) cycle. **Top:** *CHO* mutants with *HisCl1* expression in Rh6 cell, in the presence (blue) or absence (red) of Rh1 (see Fig. 5a). **Center:** Flies with downregulated HisCl1 in the Rh6 photoreceptors (red) and controls (blue) (see Fig. 4b). **Bottom:** Flies with $HisCl1^{134}$ mutant eyes (red) and controls (blue) (Fig. 4c). The average phase of the evening peak (Zeitgeber Time (ZT) of maximum locomotor activity) is calculated for every single day from 4 (top) or 2 (center, bottom) independent experiments. Errors bars represent s.e.m. (top) or the two experimental values (center, bottom).

**Genotypes**	**Number of flies n**	**Period ± s.e.m**	**Power ± s.e.m**
**Figure 1**			
Canton S	28	23.8 ± 0.14	46 ± 5
cry^{02}	21	23.8 ± 0.07	85.4 ± 7.66
$HisCl1^{134}$ ort^{1}	9	23.6 ± 0.15	73.2 ± 14.37
$HisCl1^{134}$ ort^{1} cry^{02} (*CHO*)	12	23 ± 0.07	94.9 ± 4.9
$HisCl1^{134}$ cry^{02}	16	23.3 ± 0.29	49.5 ± 5.23
ort^{1} cry^{02}	24	23.8 ± 0.13	84 ± 7.07
$ninaE^{17}$ $HisCl1^{134}$	13	23.6 ± 0.06	55 ± 7.03
$Rh6^{1}$ $HisCl1^{134}$	12	23.8 ± 0.25	53.8 ± 10.43
$ninaE^{17}$ ort^{1}	14	24.4 ± 0.13	47.2 ± 4.14
$Rh6^{1}$ ort^{1}	14	24 ± 0.17	72.5 ± 8.15
**Figure 2**			
tim-gal4/ UAS-HisCl1; CHO	16	23.6 ± 0.24	30.4 ± 3.53
tim-gal4/ UAS-ort; CHO	15	23.5 ± 0.29	23.1 ± 1.78
ort^{c1-4} -gal4/ UAS-HisCl1; CHO	14	23.2 ± 0.10	46.8 ± 6.03
ort^{c1-4} -gal4/ UAS-ort; CHO	14	23.4 ± 0.09	47.6 ± 7.67
Clk:gal4(6/1); UAS-HisCl1; CHO	16	23.1 ± 0.18	31.9 ± 4.69
Clk:gal4(6/1); UAS-ort; CHO	11	23.1 ± 0.06	103.8 ±10.54
GMR-gal4/ UAS-HisCl1; CHO	47	23.6 ± 0.06	49.4 ± 3.58
tim-gal4/ GMR-gal80; CHO, UAS-HisCl1/CHO	16	23.9 ± 0.12	81 ± 9.61
**Figure 4**			
Rh6-gal4/ UAS-HisCl1; CHO	18	23 ± 0.10	43.4 ± 3.88
Rh6-gal4/ GMR-GAL80; UAS-HisCl1, CHO	15	23.5 ± 0.07	52.4 ± 7.39
UAS-HisCl1-RNAi/+; ort^{1} cry^{02}	14	23.6 ± 0.20	55.9 ± 6.16
Rh6-gal4/ UAS-HisCl1-RNAi; ort^{1} cry^{02}	31	23.6 ± 0.10	72.8 ± 6.45
ey-gal4, UAS-flp/+; neoFRT82B, GMR-hid, l3 CL-R1, cry^{02}, ort^{1}/ neoFRT82B, cry^{02}, ort^{1} (advance)	10	23.4 ± 0.08	67.8 ± 12.67
ey-gal4, UAS-flp/+; neoFRT82B, GMR-hid, l3 CL-R1, cry^{02}, ort^{1}/ neoFRT82B, cry^{02} ort^{1} $HisCl1^{134}$ (advance)	20	23.3 ± 0.10	63.9 ± 6.70
ey-gal4, UAS-flp/+; neoFRT82B, GMR-hid, l3 CL-R1, cry^{02}, ort^{1}/ neoFRT82B, cry^{02}, ort^{1} (delay)	24	24 ± 0.34	40 ± 4.28
ey-gal4, UAS-flp/+; neoFRT82B, GMR-hid, l3 CL-R1, cry^{02}, ort^{1}/ neoFRT82B, cry^{02} ort^{1} $HisCl1^{134}$ (delay)	25	23.6 ± 0.14	41.1 ± 4.84

**Figure 5**			
Rh6-gal4/ UAS-HisCl1; CHO	30	23 ± 0.12	49.1 ± 4.04
Rh6-gal4/ UAS-HisCl1; CHO, $ninaE^{17}$	16	23.3 ± 0.07	48.8 ± 5.80
Rh6-gal4/ UAS-HisCl1; CHO, $ninaE^{17}$ $Rh6^{1}$ (red light)	17	23.3 ± 0.21	44.5 ± 2.88
so^{1}; cry^{02}	20	22 ± 0.50	40 ± 6.08
so^{1}, Rh6-gal4; CHO, UAS-HisCl1/ CHO	16	23.5 ± 0.41	34 ± 3.81
LexAop-HisCl1; Rh6-LexA/ Rh5-gal4, UAS-dti; CHO	9	23.3 ± 0.20	48.3 ± 6.04
Rh6-gal4/ UAS-HisCl1; CHO, $ninaE^{17}$ $Rh6^{1}$	21	23.1 ± 0.09	41.3 ± 3.31
$Rh5^{2}$, Rh6-gal4/ $Rh5^{2}$, UAS-HisCl1; CHO, $ninaE^{17}$ $Rh6^{1}$	16	23.3 ± 0.10	44.2 ± 4.35

**ED. Figure 3**			
Rh1-gal4/ UAS-HisCl1; CHO	20	23.2 ± 0.09	66.4 ± 6.98
Rh2-gal4/ UAS-HisCl1; CHO	24	23.2 ± 0.22	58.5 ± 8.56
Rh3-gal4/ UAS-HisCl1; CHO	15	23.3 ± 0.09	81 ± 10.89
Rh4-gal4/ UAS-HisCl1; CHO	16	22.8 ± 0.11	71 ± 8.07
Rh5-gal4/ UAS-HisCl1; CHO	16	23.9 ± 0.21	68.9 ± 5.47

Supplementary Table 1: Analysis of locomotor activity rhythms in DD. Period is given in hours ± s.e.m, with associated power (see Methods).

**One way ANOVA: Dunnett's multiple comparisons test**					
Canton S vs					
**Figure 1**	**Mean of phase**	**Mean Diff.**	**95,00% CI of diff.**	**Summary**	**Adjusted P Value**
cry^{02}	13	-0.5	-2.293 to 1.293	ns	0.9986
HisCl1^{134} *ort*^{1}	13.2	-0.7	-2.493 to 1.093	ns	0.9823
HisCl1^{134} *ort*^{1} *cry*^{02} (*CHO*)	16.3	-3.8	-5.593 to -2.007	****	<0.0001
HisCl1^{134} *cry*^{02}	12.1	0.4	-1.393 to 2.193	ns	0.999
ort^{1} *cry*^{02}	12.9	-0.4	-2.193 to 1.393	ns	0.999
ninaE^{17} *HisCl1*^{134}	12.5	0	-1.793 to 1.793	ns	>0.9999
Rh6^{1} *HisCl1*^{134}	11.6	0.9	-0.8929 to 2.693	ns	0.842
ninaE^{17} *ort*^{1}	12.1	0.4	-1.393 to 2.193	ns	0.999
Rh6^{1} *ort*^{1}	12.3	0.2	-1.593 to 1.993	ns	0.9996
**Figure 2**					
tim-gal4/ UAS-HisCl1; CHO	12.4	0.1	-1.693 to 1.893	ns	0.9998
tim-gal4/ UAS-ort; CHO	14.2	-1.7	-3.493 to 0.09289	ns	0.0759
ort^{c1-4} *-gal4/ UAS-HisCl1; CHO*	12	0.5	-1.293 to 2.293	ns	0.9986
ort^{c1-4} *-gal4 / UAS-ort; CHO*	12.5	0	-1.793 to 1.793	ns	>0.9999
Clk:gal4(6/1); UAS-HisCl1; CHO	9.5	3	1.207 to 4.793	****	<0.0001
Clk:gal4(6/1); UAS-ort; CHO	15.2	-2.7	-4.493 to -0.9071	***	0.0003
GMR-gal4/ UAS-HisCl1; CHO	12.4	0.1	-1.693 to 1.893	ns	0.9998
tim-gal4/ GMR-gal80; CHO, UAS-HisCl1/ CHO	20.6	-8.1	-9.893 to -6.307	****	<0.0001
**Figure 4**					
Rh6-gal4/ UAS-HisCl1; CHO	12	0.5	-1.293 to 2.293	ns	0.9986
Rh6-gal4/ GMR-GAL80; UAS-HisCl1, CHO	15.9	-3.4	-5.193 to -1.607	****	<0.0001
UAS-HisCl1-RNAi/+; ort^{1} *cry*^{02}	12	0.5	-1.293 to 2.293	ns	0.9986
Rh6-gal4/ UAS-HisCl1-RNAi; ort^{1} *cry*^{02}	12.6	-0.1	-1.893 to 1.693	ns	0.9998
ey-gal4, UAS-flp/+; neoFRT82B, GMR-hid, l3 CL-R1, cry^{02}*, ort*^{1}*/ neoFRT82B, cry*^{02}*, ort*^{1} (advance)	12.3	-1	-2.816 to 0.8161	ns	0.3179
ey-gal4, UAS-flp/+; neoFRT82B, GMR-hid, l3 CL-R1, cry^{02}*, ort*^{1}*/ neoFRT82B, cry*^{02} *ort*^{1} *HisCl1*^{134} (advance)	13.5	0.2	-1.616 to 2.016	ns	0.9463
ey-gal4, UAS-flp/+; neoFRT82B, GMR-hid, l3 CL-R1, cry^{02}*, ort*^{1}*/ neoFRT82B, cry*^{02}*, ort*^{1} (delay)	2.8	1.7	-2.057 to 5.457	ns	0.443
ey-gal4, UAS-flp/+; neoFRT82B, GMR-hid, l3 CL-R1, cry^{02}*, ort*^{1}*/ neoFRT82B, cry*^{02} *ort*^{1} *HisCl1*^{134} (delay)	12.2	7.7	-11.46 to -3.943	***	0.0005

**Figure 5**					
Rh6-gal4/ UAS-HisCl1; CHO	12.2	0.3	-1.493 to 2.093	ns	0.9993
Rh6-gal4/ UAS-HisCl1; CHO, ninaE^{17}	12.6	-0.1	-1.893 to 1.693	ns	0.9998
Rh6-gal4/ UAS-HisCl1; CHO, ninaE^{17} *Rh6*^{1} (red light)	8.4	4.1	2.307 to 5.893	****	<0.0001
so^{1}*; cry*^{02}	11	1.5	-0.2929 to 3.293	ns	0.1702
so^{1}*, Rh6-gal4; CHO, UAS-HisCl1/ CHO*	16.6	-4.1	-5.893 to -2.307	****	<0.0001
LexAop-HisCl1; Rh6-LexA/ Rh5-gal4, UAS-dti; CHO	12.4	0.1	-1.693 to 1.893	ns	0.9998
Rh6-gal4/ UAS-HisCl1; CHO, ninaE^{17} *Rh6*^{1}	11.9	0.6	-1.193 to 2.393	ns	0.9934
Rh5^{2}*, Rh6-gal4/ Rh5*^{2}*, UAS-HisCl1; CHO, ninaE*^{17} *Rh6*^{1}	12.1	0.4	-1.393 to 2.193	ns	0.999
**ED. Figure 3**					
Rh1-gal4/ UAS-HisCl1; CHO	14.9	-2.4	-4.474 to -0.3259	*	0.0223
Rh2-gal4/ UAS-HisCl1; CHO	16.4	-3.9	-5.974 to -1.826	***	0.0005
Rh3-gal4/ UAS-HisCl1; CHO	17.9	-5.4	-7.474 to -3.326	****	<0.0001
Rh4-gal4/ UAS-HisCl1; CHO	15.2	-2.7	-4.493 to -0.9071	***	0.0003
Rh5-gal4/ UAS-HisCl1; CHO	17.1	-4.6	-6.393 to -2.807	****	<0.0001

Supplementary Table 2: Determination of the phase of evening activity in the different genotypes and comparison with wild type flies. See Methods for phase determination and statistical analysis. ns: non-significant ($P > 0.05$), *: $P \leq 0.05$, ***: $P \leq 0.001$, **** $P \leq 0.0001$

Supplementary Methods

Fly stocks. The following lines were already described: *cry*^{02} [4], *ort*^{1} [5], *Hiscl1*^{134} [6], *ninaE*^{17} [7], *Rh5*^{2} [8], *Rh6*^{1} [9], *norpA*^{p24} [10], *so*^{1} [11], *Rh1-gal4* [12], *Rh2-gal4* [13], *Rh3-gal4* [14], *Rh4-gal4* [14], *Rh5-gal4* [15], *Rh6-gal4* [16], *Rh6*-LexA [17], ort-c$^{1\text{-}4}$-*gal4* [2], *tim-gal4* [18], *Pdf-gal4* [19], *Clk:gal4(6/1)* [20], *GMR-gal4* [21], *repo-gal4* [22], *UAS-ort* [2], *UAS-hid UAS-rpr* [23], *UAS-dti* [24], *UAS-cd8-gfp* [25], *UAS-HisCl1* RNAi [26].

Supplementary Information References

1. Hanai, S., Hamasaka, Y. & Ishida, N. Circadian entrainment to red light in Drosophila: requirement of Rhodopsin 1 and Rhodopsin 6. *Neuroreport* **19**, 1441-1444 (2008).
2. Gao, S. et al. The neural substrate of spectral preference in Drosophila. *Neuron* **60**, 328-342 (2008).
3. Pantazis, A. et al. Distinct roles for two histamine receptors (hclA and hclB) at the Drosophila photoreceptor synapse. *J Neurosci* **28**, 7250-7259 (2008).
4. Dolezelova, E., Dolezel, D. & Hall, J. C. Rhythm Defects Caused by Newly Engineered Null Mutations in Drosophila's cryptochrome Gene. *Genetics* **177**, 329-345 (2007).
5. Iovchev, M., Kodrov, P., Wolstenholme, A. J., Pak, W. L. & Semenov, E. P. Altered drug resistance and recovery from paralysis in Drosophila melanogaster with a deficient histamine-gated chloride channel. *Journal of Neurogenetics* **16**, 249-261 (2002).
6. Hong, S. T. et al. Histamine and its receptors modulate temperature-preference behaviors in Drosophila. *J Neurosci* **26**, 7245-7256 (2006).
7. O'Tousa, J. E. et al. The Drosophila ninaE gene encodes an opsin. *Cell* **40**, 839-850 (1985).
8. Yamaguchi, S., Wolf, R., Desplan, C. & Heisenberg, M. Motion vision is independent of color in Drosophila. *Proc Natl Acad Sci U S A* **105**, 4910-4915 (2008).
9. Cook, T., Pichaud, F., Sonneville, R., Papatsenko, D. & Desplan, C. Distinction between color photoreceptor cell fates is controlled by Prospero in Drosophila. *Dev Cell* **4**, 853-864 (2003).

10. Pearn, M. T., Randall, L. L., Shortridge, R. D., Burg, M. G. & Pak, W. L. Molecular, biochemical, and electrophysiological characterization of Drosophila norpA mutants. *J Biol Chem* **271**, 4937-4945 (1996).
11. Cheyette, B. N. et al. The Drosophila sine oculis locus encodes a homeodomain-containing protein required for the development of the entire visual system. *Neuron* **12**, 977-996 (1994).
12. Hardie, R. C. et al. Calcium influx via TRP channels is required to maintain PIP2 levels in Drosophila photoreceptors. *Neuron* **30**, 149-59. (2001).
13. Wernet, M. F. et al. Genetic dissection reveals two separate retinal substrates for polarization vision in Drosophila. *Curr Biol* **22**, 12-20 (2012).
14. Pichaud, F. & Desplan, C. A new visualization approach for identifying mutations that affect differentiation and organization of the Drosophila ommatidia. *Development* **128**, 815-826 (2001).
15. Mollereau, B. et al. A green fluorescent protein enhancer trap screen in drosophila photoreceptor cells. *Mech Dev* **93**, 151-160 (2000).
16. Tahayato, A. et al. Otd/Crx, a dual regulator for the specification of ommatidia subtypes in the Drosophila retina. *Dev Cell* **5**, 391-402 (2003).
17. Berger-Müller, S. et al. Assessing the role of cell-surface molecules in central synaptogenesis in the Drosophila visual system. *PLoS One* **8**, e83732 (2013).
18. Kaneko, M. Neural substrates of Drosophila rhythms revealed by mutants and molecular manipulations. *Curr Opin Neurobiol* **8**, 652-658 (1998).
19. Renn, S. C., Park, J. H., Rosbash, M., Hall, J. C. & Taghert, P. H. A pdf neuropeptide gene mutation and ablation of PDF neurons each cause severe abnormalities of behavioral circadian rhythms in Drosophila. *Cell* **99**, 791-802 (1999).
20. Gummadova, J. O., Coutts, G. A. & Glossop, N. R. Analysis of the Drosophila Clock Promoter Reveals Heterogeneity in Expression between Subgroups of Central Oscillator Cells and Identifies a Novel Enhancer Region. *J Biol Rhythms* **24**, 353-367 (2009).
21. Hay, B. A., Wolff, T. & Rubin, G. M. Expression of baculovirus P35 prevents cell death in Drosophila. *Development* **120**, 2121-9. (1994).
22. Sepp, K. J., Schulte, J. & Auld, V. J. Peripheral glia direct axon guidance across the CNS/PNS transition zone. *Dev Biol* **238**, 47-63 (2001).
23. Zhou, L. et al. Cooperative functions of the reaper and head involution defective genes in the programmed cell death of Drosophila central nervous system midline cells. *Proc Natl Acad Sci U S A* **94**, 5131-5136 (1997).

24. Han, D. D., Stein, D. & Stevens, L. M. Investigating the function of follicular subpopulations during Drosophila oogenesis through hormone-dependent enhancer-targeted cell ablation. *Development* **127**, 573-583 (2000).
25. Lee, T. & Luo, L. Mosaic analysis with a repressible neurotechnique cell marker for studies of gene function in neuronal morphogenesis. *Neuron* **22**, 451-461 (1999).
26. Oh, Y., Jang, D., Sonn, J. Y. & Choe, J. Histamine-HisCl1 receptor axis regulates wake-promoting signals in Drosophila melanogaster. *PLoS One* **8**, e68269 (2013).
